# Identifying competing endogenous RNA regulatory networks and hub genes in alcoholic liver disease for early diagnosis and potential therapeutic target insights

**DOI:** 10.18632/aging.205861

**Published:** 2024-05-24

**Authors:** Shuai-Yang Sun, Dong Hun Lee, Hao-Cheng Liu, Yi Yang, Ying-Hao Han, Taeho Kwon

**Affiliations:** 1 College of Life Science and Biotechnology, Heilongjiang Bayi Agricultural University, Daqing 163319, P.R. China; 2 Department of Biological Sciences, Research Center of Ecomimetics, Chonnam National University, Gwangju 61186, Republic of Korea; 3 Primate Resources Center, Korea Research Institute of Bioscience and Biotechnology (KRIBB), Jeonbuk 56216, Republic of Korea; 4 Department of Applied Biological Engineering, KRIBB School of Biotechnology, Korea National University of Science and Technology, Daejeon 34113, Republic of Korea

**Keywords:** alcoholic liver disease, bioinformatics, microRNA, long non-coding RNAs, competitive endogenous RNA

## Abstract

Alcoholic liver disease (ALD) has a complex pathogenesis. Although early-stage ALD can be reversed by ceasing alcohol consumption, early symptoms are difficult to detect, and several factors contribute to making alcohol difficult to quit. Continued alcohol abuse worsens the condition, meaning it may gradually progress into alcoholic hepatitis and cirrhosis, ultimately, resulting in irreversible consequences. Therefore, effective treatments are urgently needed for early-stage ALD. Current research mainly focuses on preventing the progression of alcoholic fatty liver to alcoholic hepatitis and cirrhosis. However, challenges remain in identifying key therapeutic targets and understanding the molecular mechanisms that underlie the treatment of alcoholic hepatitis and cirrhosis, such as the limited discovery of effective therapeutic targets and treatments. Here, we downloaded ALD microarray data from Gene Expression Omnibus and used bioinformatics to compare and identify the hub genes involved in the progression of alcoholic fatty liver to alcoholic hepatitis and cirrhosis. We also predicted target miRNAs and long non-coding RNAs (lncRNAs) to elucidate the regulatory mechanisms (the mRNA–miRNA–lncRNA axis) underlying this progression, thereby building a competitive endogenous RNA (ceRNA) mechanism for lncRNA, miRNA, and mRNA. This study provides a theoretical basis for the early treatment of alcoholic hepatitis and cirrhosis and identifies potential therapeutic targets.

## INTRODUCTION

Recent social progress and lifestyle changes are associated with a rising incidence of alcoholic liver disease (ALD) worldwide. ALD is mainly caused by excessive alcohol consumption and the early symptoms of liver dysfunction include hepatomegaly, liver fibrosis, and cirrhosis. If not treated early, these conditions can progress into serious conditions, including liver dysfunction and liver cell carcinoma [1]. According to the latest Global Burden of Disease Project report, released in 2016, 1.2569 million deaths were associated with cirrhosis and chronic liver disease, with alcohol consumption accounting for 27% of these cases. Furthermore, liver cancer is an important health challenge since it is associated with a mortality rate of 245,000 and accounts for 30% of all liver cancer-related deaths. Recent Chinese epidemiological surveys have shown that in some cities in the Zhejiang and Liaoning provinces, the prevalence of ALD ranges from 4.34% to 6.1% and continues to rise annually. Social progress has been associated with a rise in excessive alcohol consumption, which has increased cases of ALD. Currently, the most effective way of preventing liver disease is by abstaining from alcohol. However, only 27% of cases achieve complete recovery of the liver function, with most cases progressing into hepatitis, cirrhosis, or liver cancer [2].

ALD pathogenesis is very complex and, currently, there are no effective drugs and therapeutic targets for its treatment. Thus, there is an urgent need to identify key ALD treatment targets. Therefore, this study downloaded and analyzed publicly available sequencing data from NCBI of liver tissues from ALD patients [3]. Bioinformatics analysis was used to identify the differentially expressed genes that may be associated with the progression from alcoholic fatty liver to alcoholic hepatitis and cirrhosis. Moreover, hub genes were identified, and their mechanisms were evaluated to elucidate any potential therapeutic targets for ALD [4].

The recent increase in the use of chip technology and high-throughput sequencing [5, 6] has markedly increased the amount of information that researchers can obtain, thereby allowing improved examinations of the structure, function, and properties of organisms. For example, high throughput sequencing can measure the expression of 20,000 to 30,000 genes simultaneously [7]. Furthermore, the rapid development of second-generation sequencing technology offers a more precise analytical tool. This technique can be used to more accurately reveal the genetic characteristics of a species and identify specific genetic loci. Subsequently, researchers are able to better understand the natural environment, which has significant implications in medicine. For instance, using second-generation sequencing, researchers at the University of Nijmegen identified a Schinzel–Giedion syndrome-associated pathogenic mutation with diagnostic significance [8]. Schinzel–Giedion syndrome is a highly challenging disease with severe complications, including intellectual disability, malignant tumors, and congenital anomalies. Ultimately, using genetic sequencing [9, 10], Schinzel–Giedion syndrome can be rapidly diagnosed, which significantly shortens the time required for treatment and significantly improves patient prognoses.

In October 2021, Professor Ming He’s team used RNA sequencing to discover that SIRT2 knockout could effectively prevent alcohol-induced production of acute-phase response proteins. Subsequently, *in vivo* and *in vitro* experiments have shown that lipocalin-2 (LCN2) can effectively prevent the occurrence of ALD and modulate the antioxidant and immunoregulatory functions of SIRT2. This study identified the SIRT2–C/EBPβ–LCN2 axis as a novel regulatory mechanism underlying ALD [11], alongside its therapeutic potential against ALD. A study by Shirish Barve used 16S rRNA sequencing and whole-genome shotgun metagenomic analysis to verify a reduction in the acetyl-CoA pathway, which was induced by alcohol. Here, they identified a decrease in two key genes that encode terminal enzymes in the conversion of butanol-CoA to butyrate, yet not for (butanol-CoA: acetyl-CoA transferase) and buk (butyrate kinase), in specific taxonomic groups of butyrate-producing bacteria. Future studies should seek to identify any related bacterial species and leverage the results on the functions affected by alcohol to develop novel ALD treatment strategies [12]. Furthermore, a team led by Li-Rui Wang discovered that an alcohol-containing diet significantly downregulated the expression of the AHR gene in intestinal epithelial cells. To investigate the roles and mechanisms of this phenomenon in ALD development, the researchers developed an intestine-specific AHR knockout mouse and surprisingly, found that intestine-specific AHR knockout significantly exacerbated alcohol-induced liver injury, fat accumulation, and liver inflammation. Subsequent studies using 16S rRNA sequencing, metabolomics, and metagenomics, further identified another key therapeutic target for ALD [13].

Advances in RNA-sequencing technology have led to the discovery of a large number of miRNAs, lncRNAs, and circRNAs [14]. Indeed, miRNAs and lncRNAs are the main types of non-coding RNA in animals and plants, whereby miRNAs post-transcriptionally suppress gene expression by pairing with target mRNAs [15]. LncRNAs are a class of non-coding RNAs, which consist of more than 200 nt in length [16], and they are thought to regulate protein-coding genes in several ways. Recently, studies have indicated that lncRNAs can influence miRNA activity through several mechanisms: 1: by acting as miRNA sources; 2: by competitively interacting with miRNAs; 3: by adsorbing to miRNAs, thereby affecting gene expression [17]. Since miRNAs and lncRNAs, which are crucial for human health [18, 19], may also influence the occurrence and progression of liver diseases, they offer significant therapeutic and diagnostic potentials [20, 21].

Here, we used bioinformatics to identify the hub genes that are differentially expressed during ALD progression from mild to severe and to predict their target miRNAs and lncRNAs. Functional enrichment analysis was used to determine the roles of the predicted hub genes, miRNAs, and lncRNAs, as well as to elucidate any potential ALD therapeutic targets.

## RESULTS

### Differential gene analysis of alcoholic liver disease samples

**Figure 1:**
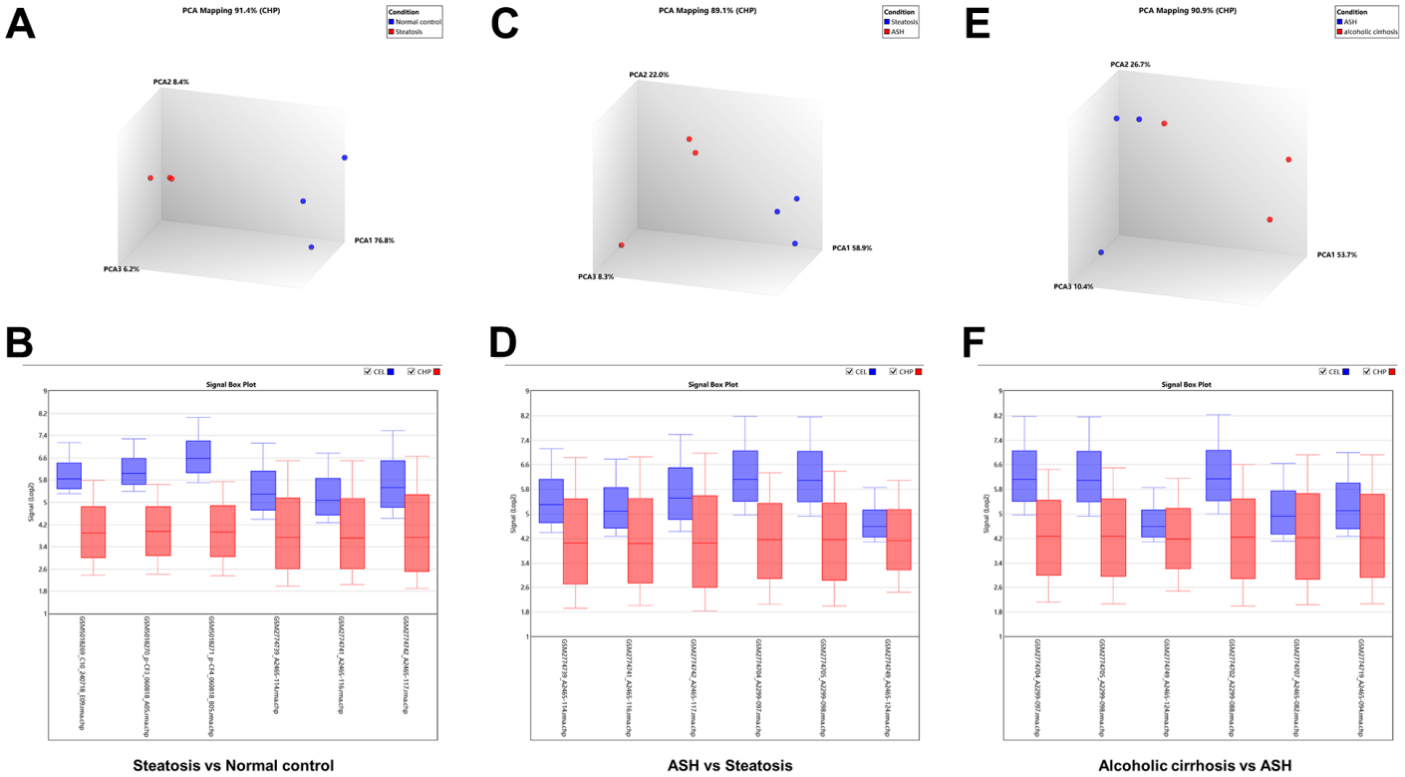
**Background correction and normalization of differential genes in alcoholic liver disease.** (**A**) PCA mapping of differential genes in alcoholic fatty liver samples compared with normal samples. (**B**) Normalization of differential genes in alcoholic fatty liver samples compared with normal samples. (**C**) PCA mapping of differential genes in alcoholic hepatitis samples compared with alcoholic fatty liver samples. (**D**) Normalization of differential genes in alcoholic hepatitis samples compared with alcoholic fatty liver samples. (**E**) PCA mapping of differential genes in alcoholic cirrhosis samples compared with alcoholic hepatitis samples. (**F**) Normalization of differential genes in alcoholic cirrhosis samples compared with alcoholic hepatitis samples.

**Figure 2:**
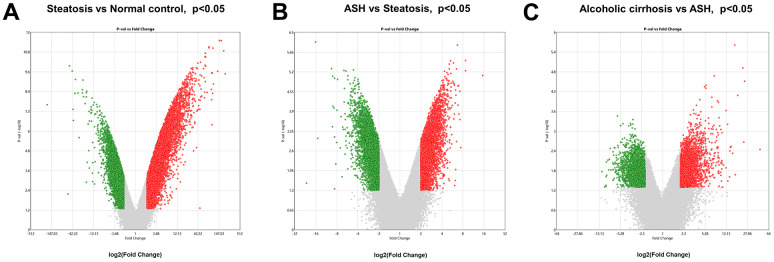
**Analysis of differential gene expression in alcoholic liver disease.** (**A**) DEGs1: Volcanic map of differential gene comparison between alcoholic fatty liver samples and normal samples. (**B**) DEGs2: Volcanic map of differential gene comparison between alcoholic hepatitis samples and alcoholic fatty liver samples. (**C**) DEGs3: Volcanic map of differential gene comparison between alcoholic cirrhosis and alcoholic hepatitis samples.

**Figure 3:**
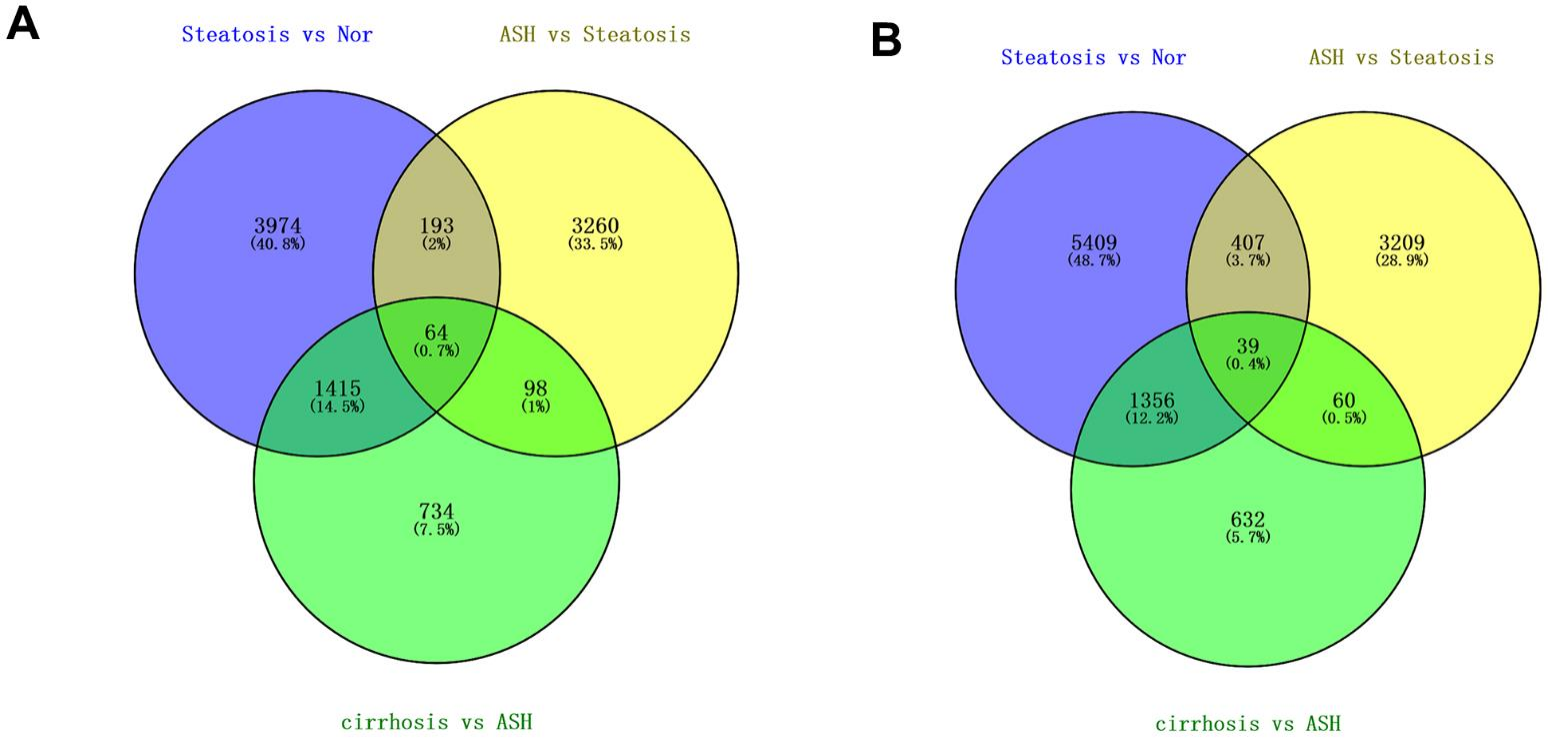
**Differentially regulated genes in all three stages of alcoholic liver disease.** (**A**) Venn diagram of up-regulated genes. (**B**) Venn diagram of down-regulated genes.

The Transcriptome Analysis Console was utilized to analyze the differentially expressed genes in the datasets GSE103580 and GSE164760. Initially, we compared the sequencing data from normal liver tissue samples with data from samples of alcoholic fatty liver. Subsequently, we compared the liver sequencing data from alcoholic fatty liver samples with data from samples of alcoholic hepatitis and alcoholic cirrhosis. Before analyzing the transcriptome data, background correction and data normalization are necessary to eliminate systematic biases and facilitate better comparisons among the samples on the same scale. TAC offers two functional modules, PCA and Signal Box Plot, to achieve this objective. PCA can conduct principal component analysis on the samples to detect and eliminate extraneous signals that could influence the outcomes. The Signal Box Plot can exhibit the box plot of each gene, identify genes with abnormal expression, and filter them out. The integration of these two methods can efficiently eliminate background signals and rectify systematic biases. We put the data of the three stages of alcoholic liver disease into sequence for processing, and obtained the PCA map and normalized data of differential gene comparison between alcoholic fatty liver and normal samples (Figure 1A, 1B), the PCA map and normalized data of differential gene comparison between alcoholic hepatitis and alcoholic fatty liver samples (Figure 1C, 1D), and the PCA maps and normalized data for differential gene alignment in alcoholic cirrhosis and alcoholic hepatitis samples (Figure 1E, 1F), respectively. Then, differentially expressed genes (DEGs1, DEGs2, DEGs3) were identified using cutoff thresholds of |Fold Change| >2 and P < 0.05. We created a scatter plot of DEGs1, with red, green, and white circles indicating up-regulated, down-regulated, and genes with no significant difference, respectively (Figure 2A). Then the scatter plots of DEGs2 and DEGs3 are drawn (Figure 2B, 2C). To identify differentially expressed genes during AMD progression, we used a Venn diagram to intersect differentially expressed genes and visualize the overlap, which resulted in 64 up-regulated genes (Figure 3A and Table 1) and 39 down-regulated genes (Figure 3B and Table 2).

**Table 1:** Upregulated differential genes at the intersection of Venn diagrams of Figure 2A.

**Name**
EIF3K
RPL28
LRRC75A-AS1
TUBA1A
ISCU
ATP6V0D1
LASP1
ERLIN2
RAB6A
AFF1
MCL1
OLA1
WBP2
RAB5C
SPARC
RAP1A
BAGE2
CSNK2B
SFPQ
TCP1
SSR1
IGHM
TNFAIP2
NUDCD3
SECISBP2
FXYD6
FXYD6-FXYD2
ANXA2
BECN1
NMI
TP53
BAG5
MYD88
BAGE
GTF2H1
GRIK1-AS2
KLF9
YME1L1
MTMR3
TAOK2
IGHG1
LIMS1
EDNRB
UBE2I
RGPD4
IGFBP5
ROCK1
ARHGAP21
NAP1L1
MTHFSD
B4GALT1
AKAP7
VCL
SUPT20H
MED25
ITM2C
C7
CTAGE15
CTAGE6
ADGRF5
SRRT
TSC1
TMEM30B
DDR1

**Table 2:** Downregulated differential genes at the intersection of Venn diagrams of Figure 2B.

**Name**
ING5
RPL28
YME1L1
ADH5
HLA-F
PPP5C
ASPH
UBE2L3
NPC2
RAP1A
SKAP2
MRPL57
AMMECR1L
ATP6V0D1
NDUFA13
CD86
LGI4
ANKS1A
OBSL1
WBP2
CLEC4M
PIGP
RFTN1
SNRPD3
SSR1
FCN2
ING1
TTI2
SMARCA2
MRPS15
PCOLCE
KLRD1
SUPT20H
SSU72
HES1
CAMK2G
TP53
NEDD8
ACADL

### Construction of protein–protein interaction network for differentially expressed genes in ALD

**Figure 4:**
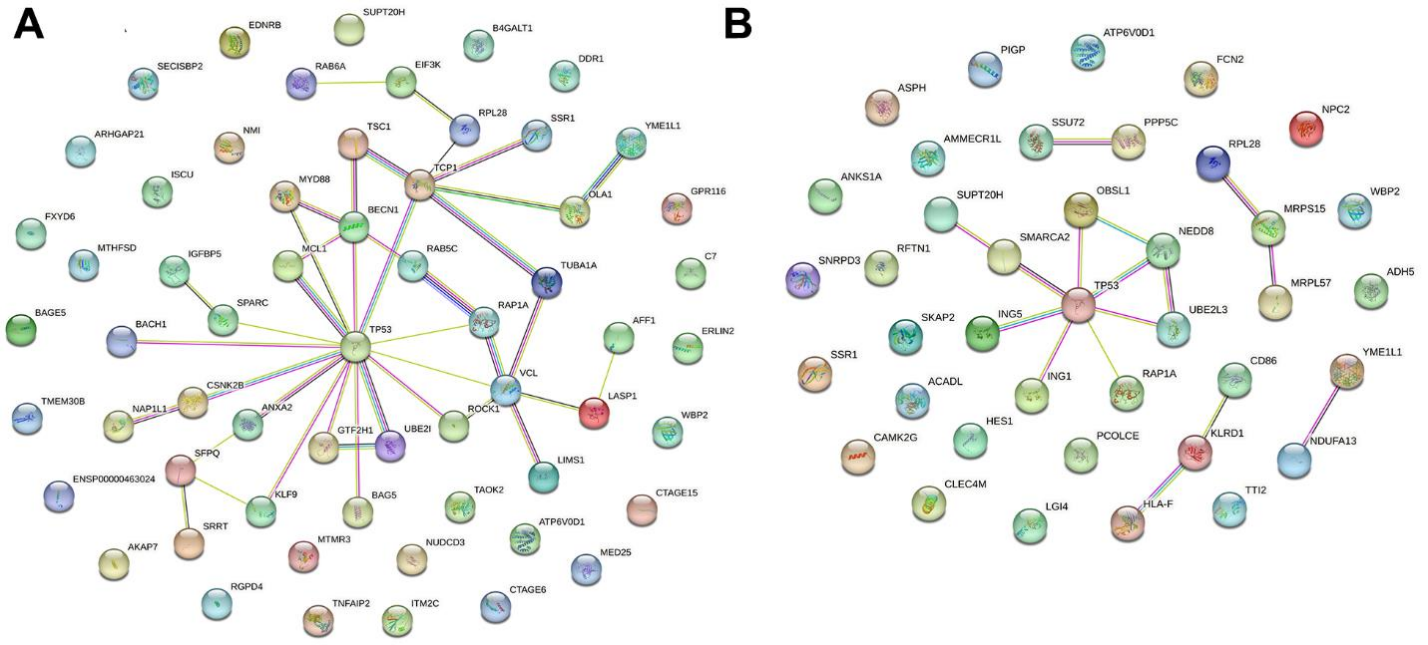
**Visualization of PPI networks in patients with alcoholic liver disease.** (**A**) Upregulated differentially expressed genes in PPI networks. (**B**) Downregulated differentially expressed genes in PPI networks. PPI network constructed by STRING.

Protein–protein interaction (PPI) network analysis is an important tool for studying the interactions and functional modules of proteins. In this study, we performed PPI network analysis using the STRING database to analyze the interactions between upregulated (Figure 4A) and downregulated (Figure 4B) differentially expressed genes and constructed the corresponding PPI networks. Analyzing the PPI networks helps to reveal the biological mechanisms underlying alcoholic liver disease, thereby facilitating the prevention and treatment of the disease.

### Screening of hub genes related to the development of ALD

**Figure 5:**
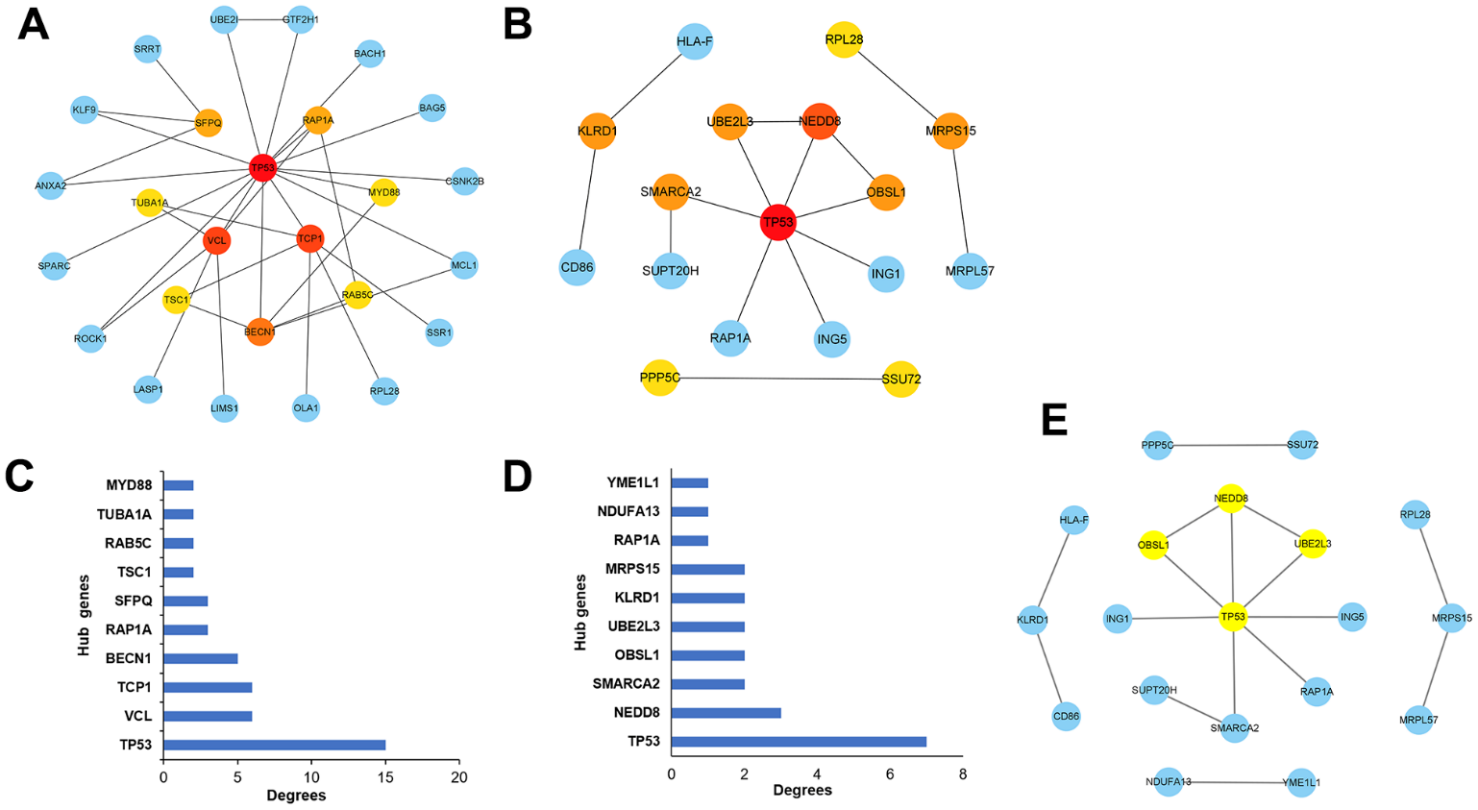
**Selection of the hub genes associated with the development of alcoholic liver disease.** (**A**) Visualizations of the hub genes associated with up-regulated differential expressed genes in the network. (**B**) Visualizations of the hub genes associated with down-regulated differential expressed genes in the network. (**C**) Bar graphs depicting the hub genes associated with up-regulated differential expressed genes. (**D**) Bar graphs depicting the hub genes associated with down-regulated differential expressed genes. (**E**) Key module analysis of the differentially expressed genes.

To identify ALD progression-associated hub genes, we constructed a PPI network of the STRING database and used the CytoHubba plugin on Cytoscape to rank genes based on degree centrality, then obtained the visual relationship diagram of up-regulated differential genes (Figure 5A) and down-regulated differential genes (Figure 5B), and selected the top 10 genes with high central correlation as the key genes. The histogram of key genes in up-regulated (Figure 5C) and down-regulated (Figure 5D) differential genes was drawn. These key genes play an important regulatory role in the study of alcoholic liver disease. Moreover, a densely connected module was identified using the MCODE plugin on Cytoscape (Figure 5E and Table 3), which can be used to further investigate their functions and interactions in-depth.

**Table 3:** Hub genes identified via network analysis are listed in Figure 5E.

**Name**	**Degree**	**Clustering Coefficient**	**MCODE: Node status**
CD86	1	0	Unclustered
KLRD1	2	0	Unclustered
HLA-F	1	0	Unclustered
ING1	1	0	Unclustered
TP53	7	0.095238	Clustered
ING5	1	0	Unclustered
MRPL57	1	0	Unclustered
MRPS15	2	0	Unclustered
RPL28	1	0	Unclustered
NDUFA13	1	0	Unclustered
YME1L1	1	0	Unclustered
NEDD8	3	0.666667	Clustered
OBSL1	2	1	Clustered
UBE2L3	2	1	Clustered
PPP5C	1	0	Unclustered
SSU72	1	0	Unclustered
RAP1A	1	0	Unclustered
SMARCA2	2	0	Unclustered
SUPT20H	1	0	Unclustered

### GO and KEGG analysis of hub genes in ALD

**Figure 6:**
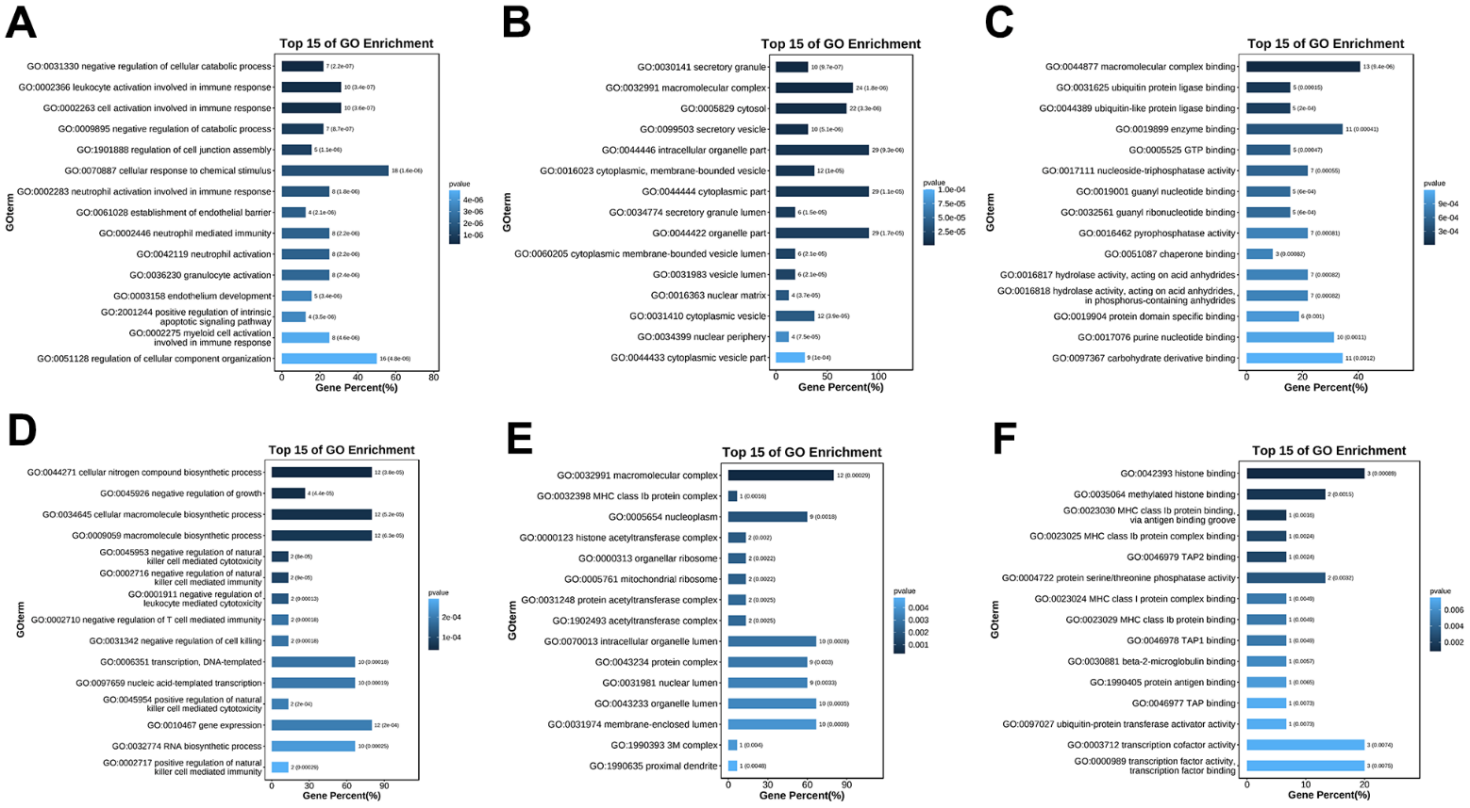
**GO enrichment analysis of the hub genes.** (**A**) GO (biological process) enrichment analysis of the upregulated differentially expressed genes. (**B**) GO (cellular component) enrichment analysis of the upregulated differentially expressed genes. (**C**) GO (molecular function) enrichment analysis of the upregulated differentially expressed genes. (**D**) GO (biological process) enrichment analysis of the downregulated differentially expressed genes. (**E**) GO (cellular component) enrichment analysis of the downregulated differentially expressed genes. (**F**) GO (molecular function) enrichment analysis of the downregulated differentially expressed genes.

**Figure 7:**
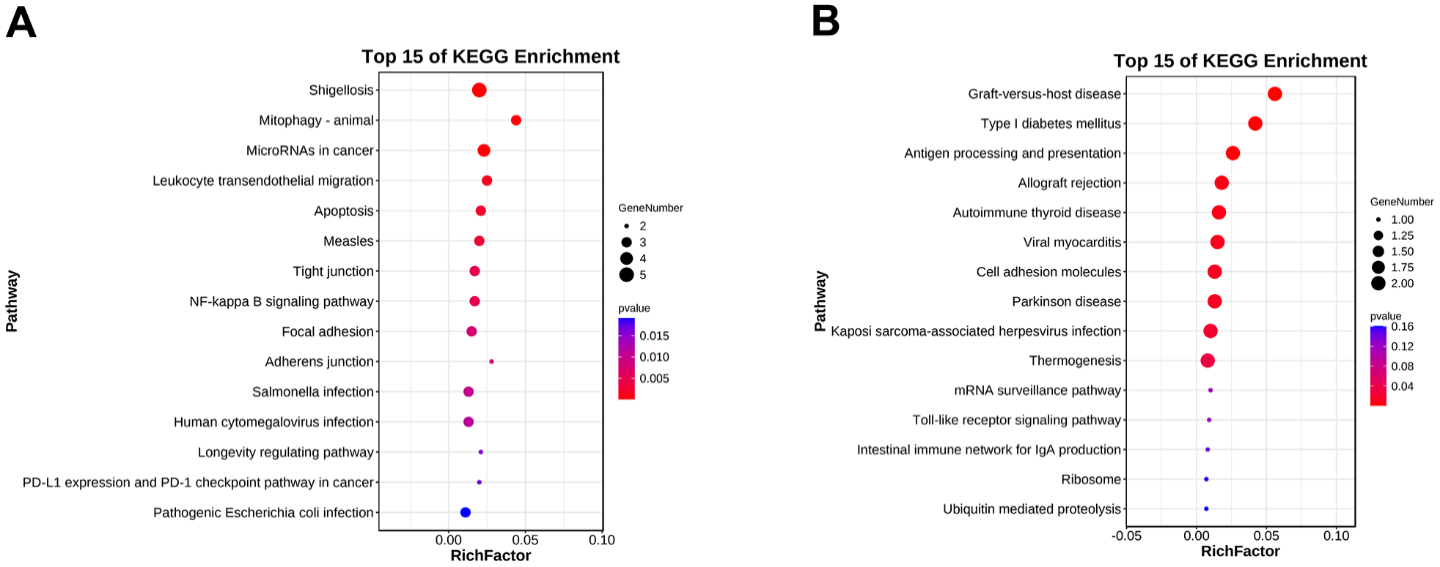
**KEGG pathway enrichment analysis of the hub genes.** (**A**) KEGG pathway enrichment analysis of the upregulated differentially expressed genes. (**B**) KEGG pathway enrichment analysis of the downregulated differentially expressed genes.

To further understand the function of hub genes, we performed GO pathway enrichment analysis of biological processes, cellular components and molecular functions. We found that the biological process of up-regulation of differential genes is mainly related to the response of cells to chemical stimulation, the regulation of cell composition and organization, and apoptosis (Figure 6A), the cellular components are mainly related to cytoplasm and organelles (Figure 6B), and the molecular functions are mainly related to the binding of macromolecular complexes and enzymes (Figure 6C). In the biological processes of down-regulating differential genes, it is mainly related to various biosynthesis and transcription (Figure 6D), in the cellular components, it is mainly related to macromolecular complex and nucleoplasm (Figure 6E), and in the molecular functions, it is mainly related to transcription factor active enzyme activity and histone binding (Figure 6F). At the same time, we also conducted enrichment analysis of KEGG pathway and found that it is related to mitochondrial autophagy and NF-κB inflammatory pathways (Figure 7A, 7B). Specifically, they are enriched in extracellular matrix formation, organelle organization, mitochondrial autophagy, and regulation of the NF-κB signaling pathway, thus indicating several potential roles for them in alcoholic liver disease.

### Construction of miRNA and lncRNA network targeting hub genes in ALD

**Figure 8:**
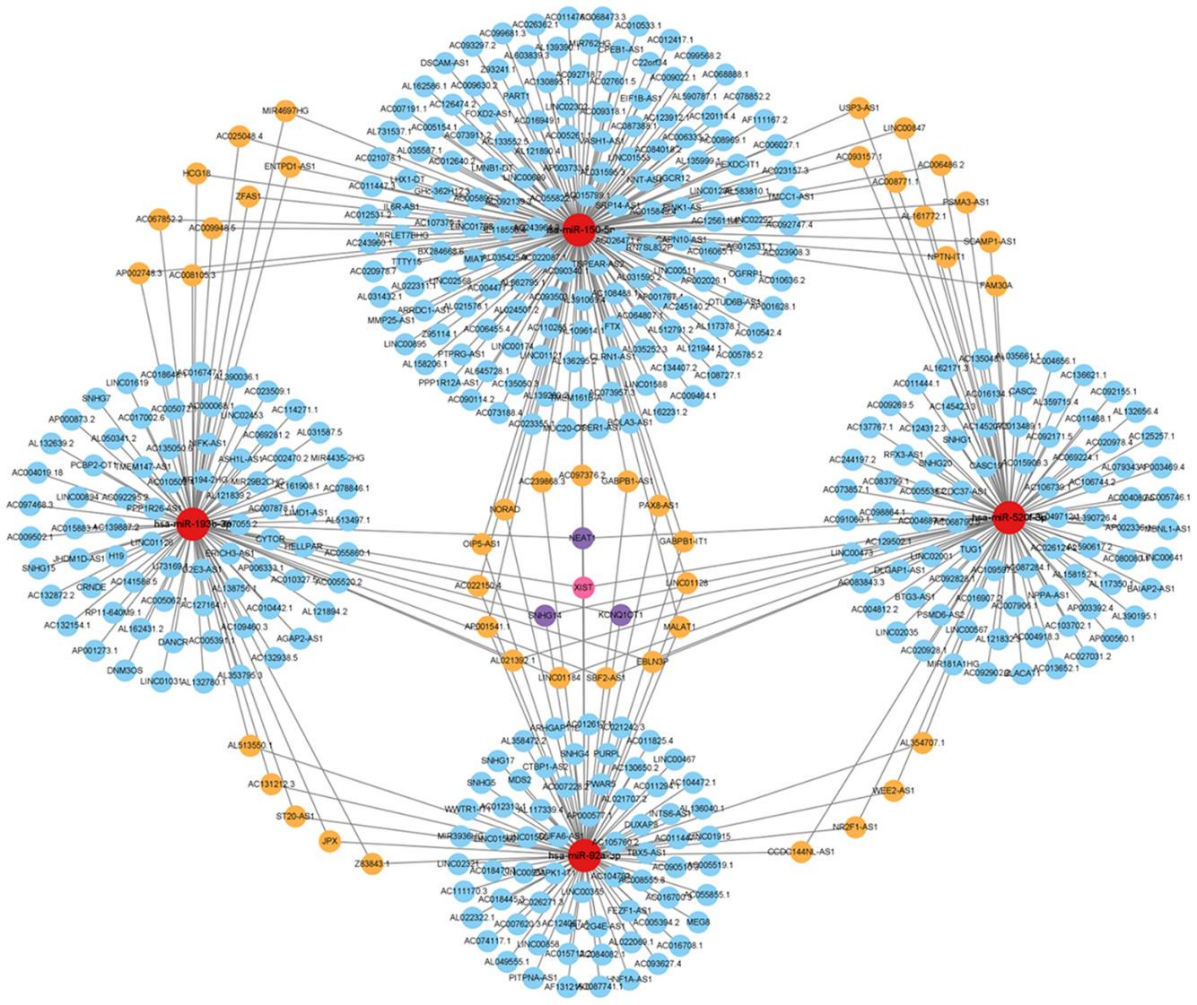
**Analysis of the hub gene-targeting miRNA–lncRNA interaction network.** miRNA–lncRNA network. Red circles represent miRNAs. Blue circles represent lncRNAs targeting one gene. Orange circles represent lncRNAs targeting two genes. Purple circles represent lncRNAs targeting three genes. Pink circles represent lncRNAs targeting four genes.

miRWalk was used to predict the miRNAs that might regulate hub gene expression. StarBase was used to conduct the interaction analysis between miRNAs and lncRNAs. The miRNA–lncRNA and miRNA–mRNA networks were constructed using Cytoscape (Figure 8 and Supplementary Tables 1, 2).

### Validation of alcoholic liver cancer using pathological tissue data

**Figure 9:**
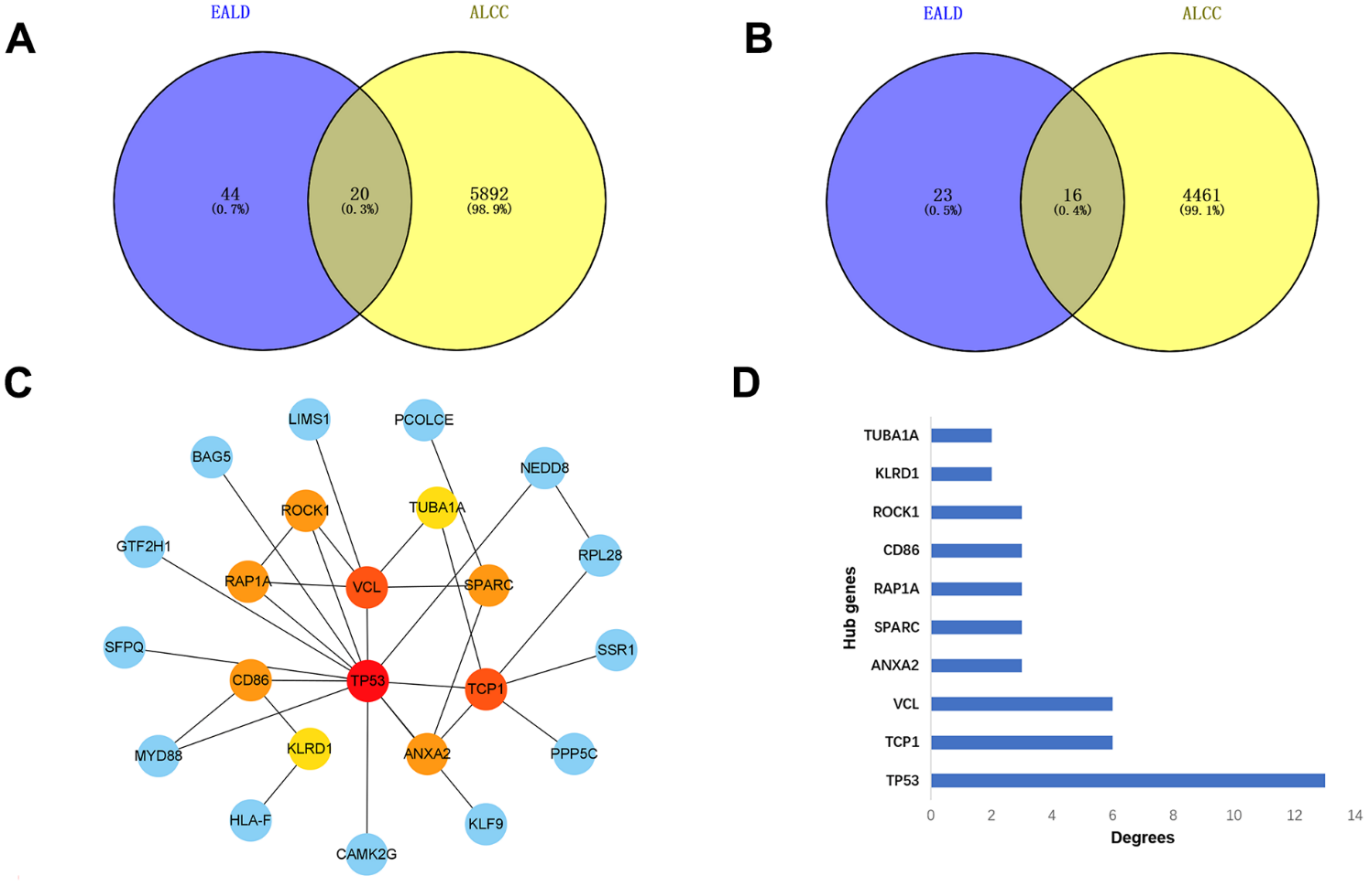
**Analysis of differential expressed genes during the four stages of alcoholic liver disease.** (**A**) Venn diagrams of up-regulated genes. (**B**) Venn diagrams of down-regulated genes. (**C**) The regulation of hub genes in all stages. (**D**) Bar graph of the hub genes.

**Figure 10:**
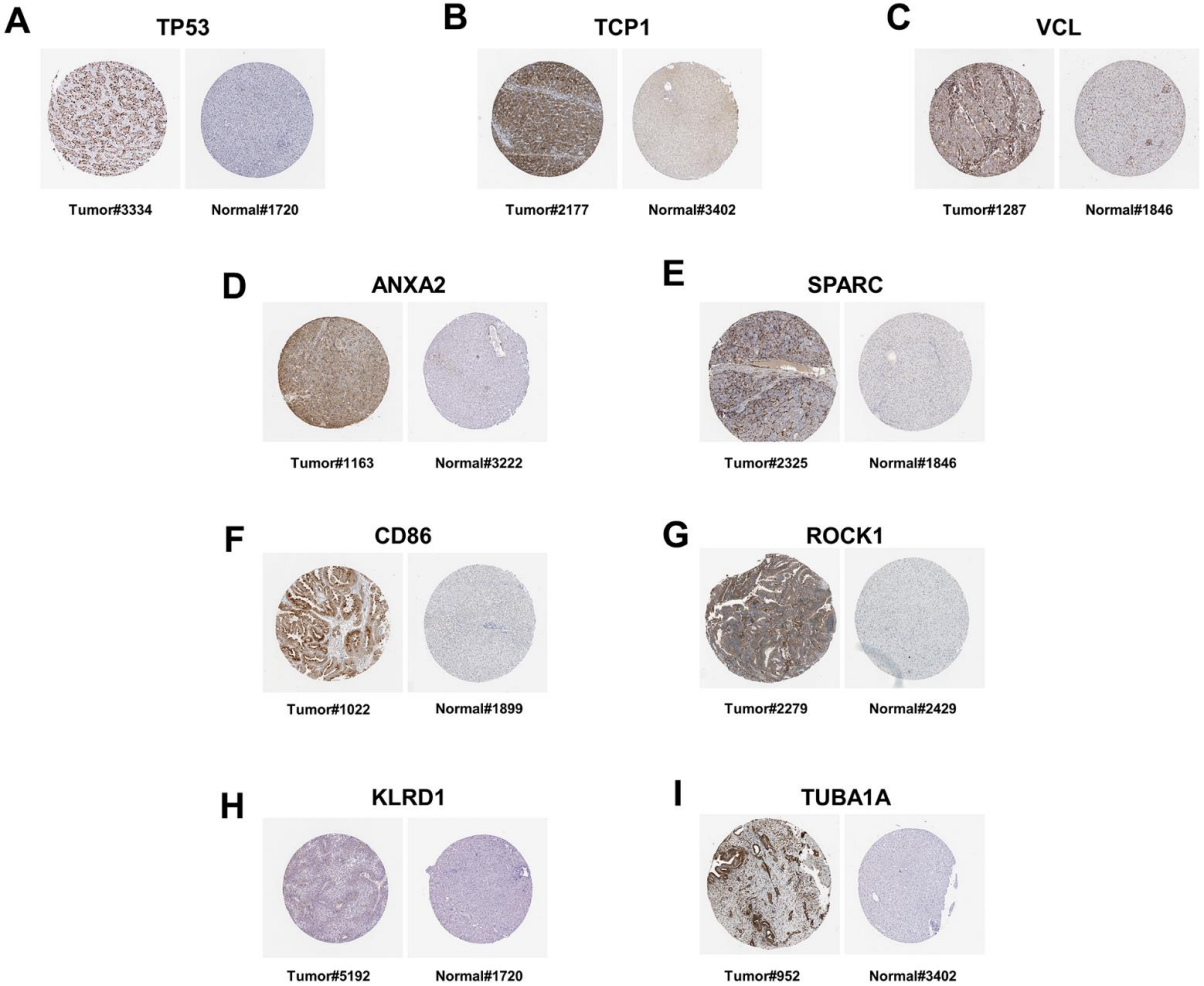
**The immunohistochemistry staining of protein in human liver tissue, as sourced from the Human Protein Atlas version 23.0.** (**A**) The immunohistochemical staining of the TP53 protein in the liver tissue of patients with liver cancer (Tumor#3334) is available from https://images.proteinatlas.org/39238/82313_B_7_4.jpg. The immunohistochemical staining of the TP53 protein in normal liver tissue (Normal#1720) is available from https://images.proteinatlas.org/2973/6986_A_8_4.jpg. (**B**) The immunohistochemical staining of the TCP1 protein in the liver tissue of patients with liver cancer (Tumor#2177) is available from https://images.proteinatlas.org/17460/45305_B_8_1.jpg. The immunohistochemical staining of the TCP1 protein in normal liver tissue (Normal#3402) is available from https://images.proteinatlas.org/27337/59054_A_7_4.jpg. (**C**) The immunohistochemical staining of the VCL protein in the liver tissue of patients with liver cancer (Tumor#1287) is available from https://images.proteinatlas.org/2131/7208_B_8_1.jpg. The immunohistochemical staining of the VCL protein in normal liver tissue (Normal#1846) is available from https://images.proteinatlas.org/2131/7209_A_8_4.jpg. (**D**) The immunohistochemical staining of the ANXA2 protein in the liver tissue of patients with liver cancer (Tumor#1163) is available from https://images.proteinatlas.org/4311/13252_B_8_5.jpg. The immunohistochemical staining of the ANXA2 protein in normal liver tissue (Normal#3222) is available from https://images.proteinatlas.org/46964/138243_A_9_4.jpg. (**E**) The immunohistochemical staining of the SPARC protein in the liver tissue of patients with liver cancer (Tumor#2325) is available from https://images.proteinatlas.org/2306/6035_B_8_6.jpg. The immunohistochemical staining of the SPARC protein in normal liver tissue (Normal#1846) is available from https://images.proteinatlas.org/2989/9780_A_7_4.jpg. (**F**) The immunohistochemical staining of the CD86 protein in the liver tissue of patients with liver cancer (Tumor#1022) is available from https://images.proteinatlas.org/4319/11662_B_8_2.jpg. The immunohistochemical staining of the CD86 protein in normal liver tissue (Normal#1899) is available from https://images.proteinatlas.org/4319/11663_A_9_4.jpg. (**G**) The immunohistochemical staining of the ROCK1 protein in the liver tissue of patients with liver cancer (Tumor#2279) is available from https://images.proteinatlas.org/7567/21572_B_7_1.jpg. The immunohistochemical staining of the ROCK1 protein in normal liver tissue (Normal#2429) is available from https://images.proteinatlas.org/7567/21573_A_8_4.jpg. (**H**) The immunohistochemical staining of the KLRD1 protein in the liver tissue of patients with liver cancer (Tumor#5192) is available from https://images.proteinatlas.org/69688/153609_B_7_1.jpg. The immunohistochemical staining of the KLRD1 protein in normal liver tissue (Normal#1720) is available from https://images.proteinatlas.org/69688/153740_A_8_4.jpg. (**I**) The immunohistochemical staining of the TUBA1A protein in the liver tissue of patients with liver cancer (Tumor#952) is available from https://images.proteinatlas.org/8686/23064_B_9_7.jpg. The immunohistochemical staining of the TUBA1A protein in normal liver tissue (Normal#3402) is available from https://images.proteinatlas.org/43684/102076_A_7_4.jpg.

To further validate the feasibility of our data, we incorporated chip data from alcoholic liver cancers in the GSE10356 dataset and compared them to data from the three stages of alcoholic liver disease (alcoholic fatty liver, alcoholic hepatitis, and alcoholic cirrhosis). From this analysis, we identified key genes that were differentially regulated in all four stages. By cross-analyzing and processing these key genes, Venn diagram of up-regulation (Figure 9A and Table 4) and down-regulation (Figure 9B and Table 5) of differential genes were obtained, and key genes that could be differential regulated at all four stages were obtained by using Cytoscape software (Figure 9C, 9D), because some key genes may activate a series of downstream genes when up-regulated, and inhibit these genes when down-regulated. So, for this part of the genetic screening, we don’t make a distinction between up and down regulation. The expression patterns of the hub genes in alcoholic liver cancer were analyzed and validated using publicly available tissue pathology data from the Human Protein Atlas (HPA). Specifically, immunohistochemical images of alcoholic liver cancer and normal liver tissue samples were obtained and examined for the expression of the identified hub genes. The data used in this study were derived from the latest version of the HPA database (version: 23.0) at the time of analysis. (Figure 10A–10I). Consistent with our previous differential expression analysis, we found that all hub genes were highly expressed in liver cancer tissues, thereby providing further evidence of their important roles in the development of alcoholic liver disease.

**Table 4:** Co-regulation differential genes at Venn diagram intersection of Figure 9A.

**Name**
TUBA1A
ATP6V0D1
ERLIN2
AFF1
SPARC
SFPQ
TCP1
TNFAIP2
ANXA2
NMI
TP53
BAG5
MYD88
GTF2H1
KLF9
LIMS1
ROCK1
B4GALT1
AKAP7
VCL

**Table 5:** Co-regulation differential genes at Venn diagram intersection of Figure 9B.

**Name**
RPL28
YME1L1
ADH5
HLA-F
PPP5C
RAP1A
SKAP2
CD86
SNRPD3
SSR1
FCN2
PCOLCE
KLRD1
HES1
CAMK2G
NEDD8

### Regulation of ceRNA mechanism in ALD

**Figure 11:**
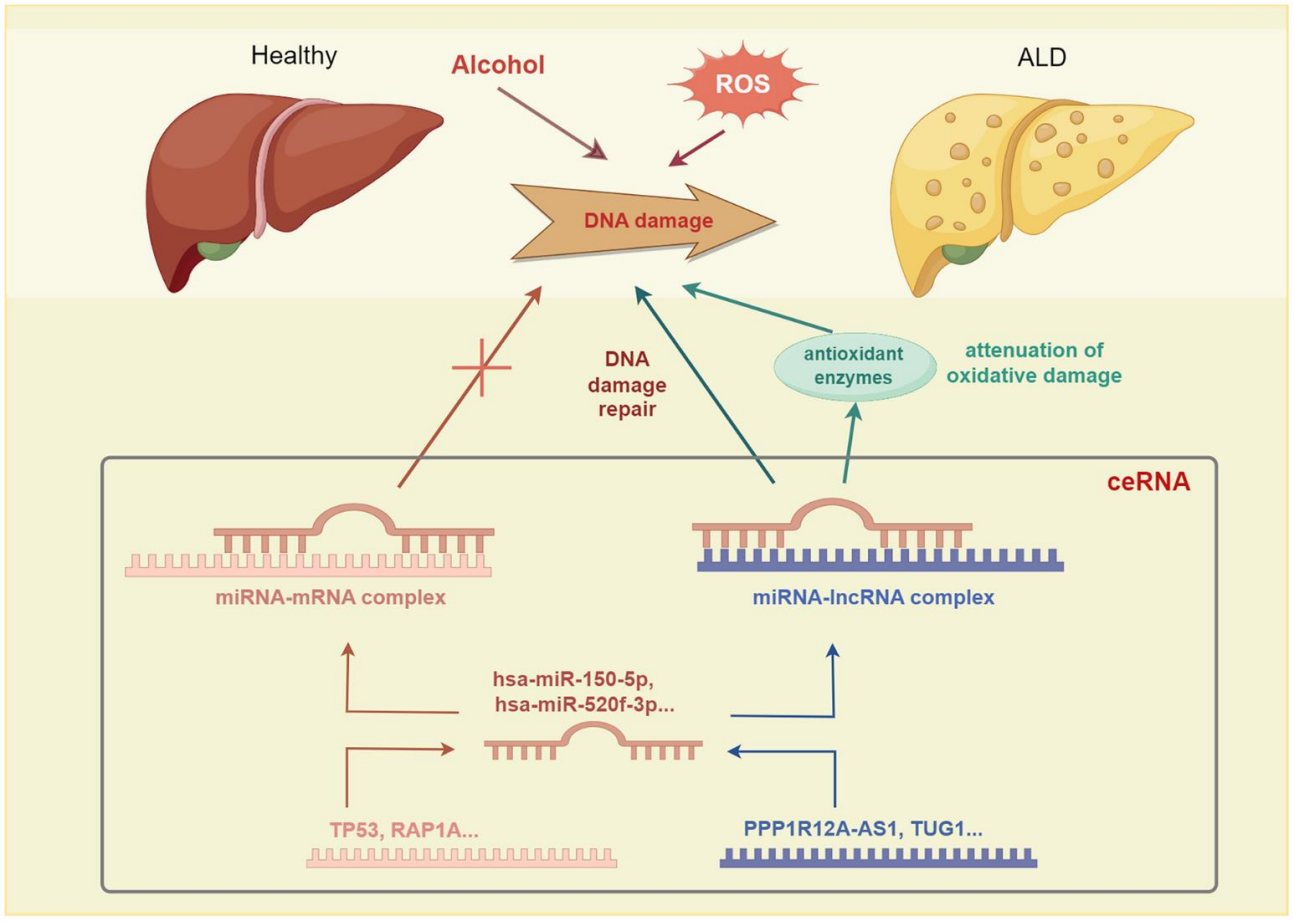
The competing endogenous RNA (ceRNA) mechanism involved has been implicated in alcoholic liver disease (ALD) pathogenesis.

Notably, lncRNA and mRNA can each act as miRNA sponges in the ceRNA network, thereby reducing miRNA binding to specific mRNAs, subsequently, protecting mRNA. Additionally, lncRNA and mRNA can mutually regulate each other by competing for miRNA binding sites, which impacts their stability and expression levels. Alcohol metabolites can cause DNA damage and oxidative stress, leading to an imbalance in cellular redox homeostasis. The TP53 mRNA can sense and respond to DNA damage signals and alleviate oxidative damage by regulating the expression of antioxidant enzymes. Moreover, hsa-miR-150-5p miRNA binds to the 3’ UTR of the TP53 gene, to form a miRNA–mRNA complex. The PPP1R12A-AS1 lncRNA can bind to hsa-miR-150-5p, thereby releasing TP53 mRNA and increasing TP53 expression. Through this ceRNA mechanism, PP1R12A-AS1 undergoes competitive binding with TP53 for miR-150-5p, thereby increasing the expression level of TP53 in the regulation of processes in alcoholic liver disease, such as DNA damage response, cell cycle, apoptosis, and oxidative stress. Similarly, TUG1 can employ the ceRNA mechanism and compete with RAP1A for binding sites in hsa-miR-520f-3p, thereby upregulating RAP1A expression, which offers potential as a treatment option for ALD (Figure 11).

## DISCUSSION

Alcoholic liver disease (ALD) is one of the most common liver diseases worldwide. Recently, the incidence of alcohol-induced liver damage has increased, including among younger age groups, especially young adults, who account for about 70% of the total cases. ALD is mainly caused by excessive alcohol-induced liver cell damage and the disruption of liver function [22]. The growing rate of alcohol abuse has contributed to the rising incidence of ALD. Both chronic and acute alcohol abuse can result in liver disease [23]. Thus, a better understanding of ALD pathogenesis, as well as effective therapeutic and diagnostic targets are urgently needed.

Multiple studies have associated ALD development with various signaling pathways, alcohol-mediated epigenetic modifications, and alterations in miRNA and lncRNA [24]. In this study, we used bioinformatics analyses to identify ALD-associated mRNAs, miRNAs, and lncRNAs, and to construct regulatory networks. We also validated their differential expressions using tissue pathology data.

By analyzing mRNA, miRNA, and lncRNA expressions in normal liver tissue, alcoholic fatty liver, alcoholic hepatitis, and alcoholic cirrhosis samples, we constructed a regulatory network and identified the potential molecular mechanisms underlying ALD development through an mRNA–miRNA–lncRNA network [25]. Analysis of the GSE103580 and GSE164760 datasets revealed that 9738 differentially expressed genes (DEGs) were upregulated and 11112 DEGs were downregulated in association with ALD. To identify the DEGs that change during the stage progressions from alcoholic fatty liver to alcoholic hepatitis (mild) and cirrhosis (severe), we analytically expressed the DEGs to a Venn diagram intersection, which identified 64 upregulated and 39 downregulated DEGs. PPI network analysis of the interaction between these DEGs and the hub genes was performed using Cytoscape. This analysis identified 10 upregulated hub genes (TP53, TCP1, VCL, TUBAIA, BECN1, RAP1A, SFPQ, TSC1, RAB5C, and MYD88) and 10 downregulated hub genes (RAP1A, KLRD1, TP53, NEDD8, SMARCA2, OBSL1, UBE2L3, MRPS15, NDUFA13, and YME1L1). Functional enrichment analysis of these hub genes revealed that they were enriched in extracellular matrix formation, cellular organelle formation, mitochondrial autophagy, and the regulation of the NF-κB signaling pathway. The hsa-miR-92a-3p, hsa-miR-150-5p, hsa-miR-193b-3p, and hsa-miR-520f-3p miRNAs were all identified as potential key factors in ALD progression, thereby highlighting their potential as biomarkers in the early diagnosis of ALD. Additionally, we predicted and analyzed target miRNAs and central regulatory genes to gain insight into the molecular functions of these dysregulated miRNAs in ALD. GO and KEGG enrichment analyses revealed that the mRNA targets of the differentially expressed miRNAs are involved in crucial biological processes, thereby highlighting their importance in major cellular processes. Pathway enrichment analysis revealed that the NF-κB and p53 signaling pathways were involved in ALD pathogenesis. These findings reveal the potential molecular mechanisms underlying ALD.

In summary, this study provides valuable insights into the roles and functions of differentially expressed miRNAs in ALD and reveals their potential involvement in ALD development and progression. Moreover, the identified hub genes are potential ALD biomarkers and form potential therapeutic targets. However, further research is needed to determine the possibility of targeting these hub genes in ALD.

The roles of TP53 (p53) in ALD are the same as in other liver diseases and cancers [26], which include DNA damage response, cell cycle regulation, apoptosis, and antioxidative stress. Metabolic byproducts, such as acetaldehyde and acetate, which are generated during alcohol metabolism can cause DNA damage. By detecting and responding to DNA damage signals, TP53 contributes to the maintenance of genomic stability. When DNA damage occurs, TP53 can prevent the proliferation of damaged cells, promote repair, or induce apoptosis to prevent tumorigenesis [27]. TP53 also plays an important role in cell cycle regulation, whereas alcohol may disrupt cell cycle progression, thereby causing abnormal cell proliferation. TP53 functions during the activation of the cell cycle checkpoint to inhibit cell cycle progression and abnormal cell proliferation. TP53 also regulates apoptosis. In ALD, impaired apoptosis may cause abnormal cell survival and liver damage. Thus, by inducing apoptosis, TP53 can eliminate abnormal cells, thereby preventing their transformation into cancer cells. TP53 can also counter alcohol-induced oxidative stress by modulating the expression of antioxidant enzymes. The byproducts of alcohol metabolism can cause oxidative stress, thereby disrupting cellular redox homeostasis [28]. TP53 can alleviate this stress by inducing the expression of antioxidant enzymes, hence, mitigating oxidative damage. Although changes in TP53 expression might indicate ALD pathogenesis, further research is needed to determine its precise mechanisms in relation to ALD.

Other genes may play important roles in the development of ALD. For example, Ras-proximate-1 (RAP1A), a member of the Ras superfamily, regulates leukocyte adhesion, extracellular matrix attachment, and the release of inflammatory factors [29]. Thus, RAP1A may regulate the interaction between liver cells and inflammatory cells in ALD, as well as modulate the intensity and extent of the inflammatory responses. RAP1A also regulates hepatic stellate cell activation and collagen deposition, thereby influencing the degree of liver fibrosis. It may also participate in the regulation of oxidative stress responses by influencing mitochondrial function, ROS production, and the expression of antioxidant enzymes. T-complex protein 1 (TCP1), also known as T-complex protein-1 ring complex (TRiC) or chaperonin containing TCP1 (CCT), is a molecular complex formed by multiple subunits, which plays a role in protein folding by influencing the unfolding and refolding of proteins. Alcohol-induced liver damage and oxidative stress may cause abnormal protein folding and degradation, thereby affecting liver cell function. Therefore, during ALD, TCP1 may be involved in protein repair and protection from damaged proteins, thereby maintaining normal liver cell function. Through comprehensive functional analyses, we have identified miRNAs and their key target genes, which have potential applications as ALD biomarkers or therapeutic targets.

In addition, although the inclusion of liver cancer data in the validation process resulted in the disappearance of some genes previously identified as hub genes, these genes can still be considered crucial genes in the early regulation of alcoholic liver disease. Despite changes in the expression of some hub genes in the liver cancer data, they can still be regarded as pivotal biomarkers in the early regulation of alcoholic liver disease. This suggests that these genes may exhibit distinct expression patterns and biological functions at different disease stages. Therefore, these genes continue to hold significant research and clinical value in the early diagnosis and treatment of alcoholic liver disease. Further investigation is warranted to elucidate the roles and regulatory mechanisms of these genes in different disease states.

In conclusion, the bioinformatic analyses conducted in this study have provided valuable insights into the roles and functions of differentially expressed miRNAs in the progression of alcoholic liver disease (ALD). The identification of four key miRNAs with regulatory roles at various stages of ALD development holds significant promise for improving the accuracy of early diagnoses of ALD. Furthermore, the identification of hub genes associated with ALD progression presents potential therapeutic targets for the treatment of ALD.

The differential gene expression analysis revealed a comprehensive understanding of the molecular mechanisms underlying ALD, shedding light on the potential roles of TP53, RAP1A, and TCP1 in ALD pathogenesis. These findings have the potential to significantly impact the development of diagnostic and therapeutic strategies for ALD. The functional enrichment analysis of the hub genes has highlighted their involvement in crucial biological processes, cellular components, and molecular functions, providing a deeper understanding of their roles in ALD.

Moreover, the construction of the miRNA–lncRNA network targeting hub genes has unveiled potential regulatory mechanisms underlying ALD, offering new avenues for further research and potential therapeutic interventions. The validation of hub gene expression using tissue pathology data has further strengthened the credibility of the findings, emphasizing the importance of these hub genes in the development of ALD.

Overall, the findings of this study have the potential to advance the field of ALD research by providing a foundation for the development of novel diagnostic biomarkers and therapeutic targets. Further research and validation of the identified miRNAs and hub genes are warranted to fully elucidate their roles in ALD pathogenesis and to determine their potential as targets for therapeutic intervention. Further research is needed to fully establish the therapeutic target value of these key genes in ALD.

## MATERIALS AND METHODS

### Raw data analysis

The publicly available gene expression datasets, GSE103580 and GSE164760, were downloaded from Gene Expression Omnibus. The GSE103580 dataset contains gene expression sequences from liver samples of three patients with alcoholic fatty livers (GSM2774739, GSM2774741, and GSM2774742), three patients with alcoholic hepatitis (GSM2774704, GSM2774705, and GSM2774749), three patients with alcoholic cirrhosis (GSM2774703, GSM2774707, and GSM2774709), and three normal liver samples (GSM5018269, GSM5018270, and GSM5018271).

### Identification of differentially expressed genes

Using the transcriptome analysis console, we compared liver gene expression sequences from the alcoholic fatty liver samples and alcoholic cirrhosis samples in dataset GSE103580. We used appropriate genetic testing methods to determine the differences between them. Results from the genomic testing revealed significant differences in the detected genomic conclusions (P < 0.05). Next, we used Venny 2.1 (https://bioinfogp.cnb.csic.es/tools/venny/index.html) to identify and visualize the intersections between the upregulated and downregulated genes.

### Protein–protein interaction (PPI) network construction and module analysis

Using STRING (https://cn.string-db.org/), a PPI network was constructed from differentially expressed genes and then visualized using Cytoscape V.3.9.0. Moreover, after careful selection and using the Cytoscape MCODE plugin, we successfully classified the relevant PPI network modules based on the criteria, K-core = 2, degree cutoff = 2, max depth = 100, and node score cutoff = 0.2.

### Hub gene selection and analysis

Using the CytoHubba plugin on Cytoscape, we calculated the degree centrality of the protein nodes and identified the hub genes using a degree cutoff threshold of ≥10. Next, analysis of OmicShare genomic data and visualization platform (https://www.omicshare.com/index.php) was performed for the Gene Ontology (GO) and Kyoto Encyclopedia of Genes and Genomes (KEGG) pathway enrichment analysis of the hub genes. P ≤ 0.05 indicated statistical significance.

### Prediction of hub gene-targeting miRNAs and lncRNAs

miRWalk 3.0 (http://mirwalk.umm.uni-heidelberg.de/), which is a tool that integrates the prediction results from TargetScan and miRDB, with a threshold of ≥ 0.95 for miRWalk prediction analysis, was used to predict interactions between miRNAs and hub genes. Finally, Cytoscape was used to construct a miRNA–mRNA network. StarBase (https://rnasysu.com/encori/index.php) was used to predict interactions between lncRNAs and hub genes, followed by Cytoscape analysis to construct a miRNA–lncRNA network.

### Validation of ALD pathological data

The expression patterns of the hub genes in alcoholic liver cancer were examined on the HPA (https://www.proteinatlas.org/) website. Publicly available data on tissue pathology were used to validate the hub gene expression results.

### Data availability

The public datasets to support the results of this subject can be gained from NCBI (https://www.ncbi.nlm.nih.gov/), STRING (https://cn.string-db.org/), OmicShare (https://www.omicshare.com/index.php), miRWalk 3.0 (http://mirwalk.umm.uni-heidelberg.de/), StarBase (https://rnasysu.com/encori/index.php), and HPA (https://www.proteinatlas.org/).

## Supplementary Material

Supplementary Table 1

Supplementary Table 2
