# Supplementary material for: Identifying competing endogenous RNA regulatory networks and hub genes in alcoholic liver disease for early diagnosis and potential therapeutic target insights
Source: Aging (Albany NY). 2024 May 24;16(10):9147–67. doi: 10.18632/aging.205861 (PMC11164510; doi:10.18632/aging.205861)
Supplement: Supplementary Table 1 [file aging-16-205861-s001.pdf]

SUPPLEMENTARY TABLE

Supplementary Table 1. Co-regulation differential genes at Venn diagram intersection of Figure 8.

Name
RPL28
TUBA1A
ATP6V0D1
LASP1
ERLIN2
RAB6A
AFF1
MCL1
RAB5C
SPARC
RAP1A
CSNK2B
SFPQ
TCP1
SSR1
IGHM
TNFAIP2
ANXA2
BECN1
NMI
TP53
BAG5
MYD88
BAGE
GTF2H1
KLF9
YME1L1
MTMR3
TAOK2
IGHG1
LIMS1
EDNRB
UBE2I
IGFBP5
ROCK1
NAP1L1
B4GALT1
AKAP7
VCL
C7
TSC1
DDR1

ADH5
HLA-F
PPP5C
ASPH
UBE2L3
NPC2
SKAP2
CD86
CLEC4M
SNRPD3
FCN2
ING1
SMARCA2
PCOLCE
KLRD1
HES1
CAMK2G
NEDD8
ACADL
