# Supplementary material for: Identifying competing endogenous RNA regulatory networks and hub genes in alcoholic liver disease for early diagnosis and potential therapeutic target insights
Source: Aging (Albany NY). 2024 May 24;16(10):9147–67. doi: 10.18632/aging.205861 (PMC11164510; doi:10.18632/aging.205861)
Supplement: Supplementary Table 2 [file aging-16-205861-s002.docx]

**Supplementary Table 1. Hub gene target miRNA and lncRNA network of Figure 8A.**

| Name | Type | Degree | BetweennessCentrality |
| --- | --- | --- | --- |
| hsa-miR-92a-3p | miRNA | 89 | 0.308103 |
| hsa-miR-150-5p | miRNA | 187 | 0.629198 |
| hsa-miR-193b-3p | miRNA | 97 | 0.341241 |
| hsa-miR-520f-3p | miRNA | 104 | 0.366974 |
| LINC01128 | lncRNA | 2 | 0.011052 |
| MDS2 | lncRNA | 1 | 0 |
| AC239868.3 | lncRNA | 2 | 0.011052 |
| AL358472.2 | lncRNA | 1 | 0 |
| LINC00467 | lncRNA | 1 | 0 |
| AC074117.1 | lncRNA | 1 | 0 |
| AC016700.3 | lncRNA | 1 | 0 |
| PAX8-AS1 | lncRNA | 2 | 0.011052 |
| AC018470.1 | lncRNA | 1 | 0 |
| AC016708.1 | lncRNA | 1 | 0 |
| AC105760.2 | lncRNA | 1 | 0 |
| WWTR1-IT1 | lncRNA | 1 | 0 |
| AC104472.1 | lncRNA | 1 | 0 |
| AC007620.3 | lncRNA | 1 | 0 |
| CTBP1-AS2 | lncRNA | 1 | 0 |
| AC097376.2 | lncRNA | 2 | 0.011052 |
| AC104793.1 | lncRNA | 1 | 0 |
| PURPL | lncRNA | 1 | 0 |
| NR2F1-AS1 | lncRNA | 2 | 0.010158 |
| MIR3936HG | lncRNA | 1 | 0 |
| SNHG4 | lncRNA | 1 | 0 |
| AL049555.1 | lncRNA | 1 | 0 |
| SNHG5 | lncRNA | 1 | 0 |
| AL513550.1 | lncRNA | 2 | 0.00823 |
| AL022069.1 | lncRNA | 1 | 0 |
| AC093627.4 | lncRNA | 1 | 0 |
| AC011294.1 | lncRNA | 1 | 0 |
| FEZF1-AS1 | lncRNA | 1 | 0 |
| WEE2-AS1 | lncRNA | 2 | 0.010158 |
| AC021242.3 | lncRNA | 1 | 0 |
| AF131215.6 | lncRNA | 1 | 0 |
| AC124067.4 | lncRNA | 1 | 0 |
| AC084082.1 | lncRNA | 1 | 0 |
| AL354707.1 | lncRNA | 2 | 0.010158 |
| DAPK1-IT1 | lncRNA | 1 | 0 |
| LINC00963 | lncRNA | 1 | 0 |
| AL117339.4 | lncRNA | 1 | 0 |
| LINC00858 | lncRNA | 1 | 0 |
| KCNQ1OT1 | lncRNA | 3 | 0.028399 |
| MALAT1 | lncRNA | 2 | 0.011052 |
| AP000577.1 | lncRNA | 1 | 0 |
| AP001541.1 | lncRNA | 2 | 0.011052 |
| TBX5-AS1 | lncRNA | 1 | 0 |
| HNF1A-AS1 | lncRNA | 1 | 0 |
| AC131212.3 | lncRNA | 2 | 0.00823 |
| LINC00365 | lncRNA | 1 | 0 |
| INTS6-AS1 | lncRNA | 1 | 0 |
| AC005519.1 | lncRNA | 1 | 0 |
| AL136040.1 | lncRNA | 1 | 0 |
| LINC02321 | lncRNA | 1 | 0 |
| LINC01550 | lncRNA | 1 | 0 |
| MEG8 | lncRNA | 1 | 0 |
| PWAR5 | lncRNA | 1 | 0 |
| SNHG14 | lncRNA | 3 | 0.028399 |
| ARHGAP11B | lncRNA | 1 | 0 |
| OIP5-AS1 | lncRNA | 2 | 0.011052 |
| PLA2G4E-AS1 | lncRNA | 1 | 0 |
| AC090510.3 | lncRNA | 1 | 0 |
| GABPB1-IT1 | lncRNA | 2 | 0.011052 |
| GABPB1-AS1 | lncRNA | 2 | 0.011052 |
| AC055855.1 | lncRNA | 1 | 0 |
| ST20-AS1 | lncRNA | 2 | 0.00823 |
| AC015712.2 | lncRNA | 1 | 0 |
| AC130650.2 | lncRNA | 1 | 0 |
| PITPNA-AS1 | lncRNA | 1 | 0 |
| AC026271.3 | lncRNA | 1 | 0 |
| CCDC144NL-AS1 | lncRNA | 2 | 0.010158 |
| AC111170.3 | lncRNA | 1 | 0 |
| AC087741.1 | lncRNA | 1 | 0 |
| LINC01915 | lncRNA | 1 | 0 |
| AC011825.4 | lncRNA | 1 | 0 |
| AC018445.3 | lncRNA | 1 | 0 |
| AC011447.7 | lncRNA | 1 | 0 |
| AC005394.2 | lncRNA | 1 | 0 |
| AC008555.8 | lncRNA | 1 | 0 |
| AC012617.1 | lncRNA | 1 | 0 |
| AC022150.4 | lncRNA | 2 | 0.011052 |
| AC007228.2 | lncRNA | 1 | 0 |
| AC012313.1 | lncRNA | 1 | 0 |
| NORAD | lncRNA | 2 | 0.011052 |
| SNHG17 | lncRNA | 1 | 0 |
| DUXAP8 | lncRNA | 1 | 0 |
| AL022322.1 | lncRNA | 1 | 0 |
| AL021707.2 | lncRNA | 1 | 0 |
| NDUFA6-AS1 | lncRNA | 1 | 0 |
| LINC01560 | lncRNA | 1 | 0 |
| XIST | lncRNA | 4 | 0.066402 |
| JPX | lncRNA | 2 | 0.00823 |
| Z83843.1 | lncRNA | 2 | 0.00823 |
| AL645728.1 | lncRNA | 1 | 0 |
| BX284668.6 | lncRNA | 1 | 0 |
| PINK1-AS | lncRNA | 1 | 0 |
| AL031432.1 | lncRNA | 1 | 0 |
| AL603839.3 | lncRNA | 1 | 0 |
| AL139289.2 | lncRNA | 1 | 0 |
| FOXD2-AS1 | lncRNA | 1 | 0 |
| AC099568.2 | lncRNA | 1 | 0 |
| AC093157.1 | lncRNA | 2 | 0.013444 |
| AL391069.4 | lncRNA | 1 | 0 |
| IL6R-AS1 | lncRNA | 1 | 0 |
| AC108488.1 | lncRNA | 1 | 0 |
| RN7SL832P | lncRNA | 1 | 0 |
| LINC01121 | lncRNA | 1 | 0 |
| LINC01798 | lncRNA | 1 | 0 |
| BOLA3-AS1 | lncRNA | 1 | 0 |
| AC009948.5 | lncRNA | 2 | 0.013507 |
| CAPN10-AS1 | lncRNA | 1 | 0 |
| EIF1B-AS1 | lncRNA | 1 | 0 |
| PTPRG-AS1 | lncRNA | 1 | 0 |
| TMCC1-AS1 | lncRNA | 1 | 0 |
| AC108727.1 | lncRNA | 1 | 0 |
| CLRN1-AS1 | lncRNA | 1 | 0 |
| MUC20-OT1 | lncRNA | 1 | 0 |
| AC016949.1 | lncRNA | 1 | 0 |
| AC078852.2 | lncRNA | 1 | 0 |
| AP002026.1 | lncRNA | 1 | 0 |
| AC012640.2 | lncRNA | 1 | 0 |
| NNT-AS1 | lncRNA | 1 | 0 |
| AC093297.2 | lncRNA | 1 | 0 |
| PART1 | lncRNA | 1 | 0 |
| SCAMP1-AS1 | lncRNA | 2 | 0.013444 |
| AC008771.1 | lncRNA | 2 | 0.013444 |
| TMEM161B-AS1 | lncRNA | 1 | 0 |
| LMNB1-DT | lncRNA | 1 | 0 |
| AC021078.1 | lncRNA | 1 | 0 |
| LINC00847 | lncRNA | 2 | 0.013444 |
| AL139390.1 | lncRNA | 1 | 0 |
| AL121944.1 | lncRNA | 1 | 0 |
| AL662795.1 | lncRNA | 1 | 0 |
| HCG18 | lncRNA | 2 | 0.013507 |
| AL035587.1 | lncRNA | 1 | 0 |
| AL024507.2 | lncRNA | 1 | 0 |
| AL117378.1 | lncRNA | 1 | 0 |
| AC073957.3 | lncRNA | 1 | 0 |
| AC006027.1 | lncRNA | 1 | 0 |
| AC005154.1 | lncRNA | 1 | 0 |
| AC099681.3 | lncRNA | 1 | 0 |
| AC006455.4 | lncRNA | 1 | 0 |
| AC073188.4 | lncRNA | 1 | 0 |
| LINC00174 | lncRNA | 1 | 0 |
| AC006333.2 | lncRNA | 1 | 0 |
| AC090114.2 | lncRNA | 1 | 0 |
| LINC00689 | lncRNA | 1 | 0 |
| AC016065.1 | lncRNA | 1 | 0 |
| AC009630.2 | lncRNA | 1 | 0 |
| AC064807.1 | lncRNA | 1 | 0 |
| AC055822.1 | lncRNA | 1 | 0 |
| OTUD6B-AS1 | lncRNA | 1 | 0 |
| AC107375.1 | lncRNA | 1 | 0 |
| AL158206.1 | lncRNA | 1 | 0 |
| AL162231.2 | lncRNA | 1 | 0 |
| AL162586.1 | lncRNA | 1 | 0 |
| ARRDC1-AS1 | lncRNA | 1 | 0 |
| AL731537.1 | lncRNA | 1 | 0 |
| LINC01553 | lncRNA | 1 | 0 |
| ENTPD1-AS1 | lncRNA | 2 | 0.013507 |
| AP003733.4 | lncRNA | 1 | 0 |
| NEAT1 | lncRNA | 3 | 0.036962 |
| AP002748.3 | lncRNA | 2 | 0.013507 |
| AP001767.4 | lncRNA | 1 | 0 |
| MIR4697HG | lncRNA | 2 | 0.013507 |
| AC092747.4 | lncRNA | 1 | 0 |
| AC009318.1 | lncRNA | 1 | 0 |
| AC023157.3 | lncRNA | 1 | 0 |
| AC009464.1 | lncRNA | 1 | 0 |
| AC125611.4 | lncRNA | 1 | 0 |
| AC068888.1 | lncRNA | 1 | 0 |
| AC012531.2 | lncRNA | 1 | 0 |
| AC012531.1 | lncRNA | 1 | 0 |
| PPP1R12A-AS1 | lncRNA | 1 | 0 |
| AC126474.2 | lncRNA | 1 | 0 |
| AC130895.1 | lncRNA | 1 | 0 |
| AC084018.2 | lncRNA | 1 | 0 |
| AC026362.1 | lncRNA | 1 | 0 |
| AC073911.2 | lncRNA | 1 | 0 |
| AL161772.1 | lncRNA | 2 | 0.013444 |
| AL590787.1 | lncRNA | 1 | 0 |
| AL135999.1 | lncRNA | 1 | 0 |
| AL136295.2 | lncRNA | 1 | 0 |
| LINC02302 | lncRNA | 1 | 0 |
| LINC01588 | lncRNA | 1 | 0 |
| PSMA3-AS1 | lncRNA | 2 | 0.013444 |
| AF111167.2 | lncRNA | 1 | 0 |
| VASH1-AS1 | lncRNA | 1 | 0 |
| AL512791.2 | lncRNA | 1 | 0 |
| LINC02292 | lncRNA | 1 | 0 |
| AL118558.4 | lncRNA | 1 | 0 |
| AL583810.1 | lncRNA | 1 | 0 |
| FAM30A | lncRNA | 2 | 0.013444 |
| AC023908.3 | lncRNA | 1 | 0 |
| SRP14-AS1 | lncRNA | 1 | 0 |
| AC023355.1 | lncRNA | 1 | 0 |
| AC022087.1 | lncRNA | 1 | 0 |
| LINC02568 | lncRNA | 1 | 0 |
| USP3-AS1 | lncRNA | 2 | 0.013444 |
| NPTN-IT1 | lncRNA | 2 | 0.013444 |
| CPEB1-AS1 | lncRNA | 1 | 0 |
| MMP25-AS1 | lncRNA | 1 | 0 |
| AC133552.5 | lncRNA | 1 | 0 |
| AC120114.4 | lncRNA | 1 | 0 |
| MIR762HG | lncRNA | 1 | 0 |
| AC135050.3 | lncRNA | 1 | 0 |
| AC026471.6 | lncRNA | 1 | 0 |
| AC010533.1 | lncRNA | 1 | 0 |
| AC010542.4 | lncRNA | 1 | 0 |
| AC020978.7 | lncRNA | 1 | 0 |
| AC009022.1 | lncRNA | 1 | 0 |
| AC092718.7 | lncRNA | 1 | 0 |
| AC092139.3 | lncRNA | 1 | 0 |
| AC015799.1 | lncRNA | 1 | 0 |
| AC087388.1 | lncRNA | 1 | 0 |
| AC005899.3 | lncRNA | 1 | 0 |
| AC015849.4 | lncRNA | 1 | 0 |
| LHX1-DT | lncRNA | 1 | 0 |
| AC067852.2 | lncRNA | 2 | 0.013507 |
| AC008105.3 | lncRNA | 2 | 0.013507 |
| AC004477.1 | lncRNA | 1 | 0 |
| AC025048.4 | lncRNA | 2 | 0.013507 |
| AC134407.2 | lncRNA | 1 | 0 |
| LINC00511 | lncRNA | 1 | 0 |
| AC027601.5 | lncRNA | 1 | 0 |
| AC110285.2 | lncRNA | 1 | 0 |
| HEXDC-IT1 | lncRNA | 1 | 0 |
| AC012417.1 | lncRNA | 1 | 0 |
| AC090340.1 | lncRNA | 1 | 0 |
| AC068473.3 | lncRNA | 1 | 0 |
| AC005785.2 | lncRNA | 1 | 0 |
| AC011447.3 | lncRNA | 1 | 0 |
| AC010636.2 | lncRNA | 1 | 0 |
| AC123912.1 | lncRNA | 1 | 0 |
| AC243960.1 | lncRNA | 1 | 0 |
| AC006486.2 | lncRNA | 2 | 0.013444 |
| AC243964.3 | lncRNA | 1 | 0 |
| AC007191.1 | lncRNA | 1 | 0 |
| AC093503.1 | lncRNA | 1 | 0 |
| AC011476.3 | lncRNA | 1 | 0 |
| AC005261.1 | lncRNA | 1 | 0 |
| AC008969.1 | lncRNA | 1 | 0 |
| AL121890.4 | lncRNA | 1 | 0 |
| AL035252.3 | lncRNA | 1 | 0 |
| AL109614.1 | lncRNA | 1 | 0 |
| OSER1-AS1 | lncRNA | 1 | 0 |
| AL021578.1 | lncRNA | 1 | 0 |
| ZFAS1 | lncRNA | 2 | 0.013507 |
| DSCAM-AS1 | lncRNA | 1 | 0 |
| AP001628.1 | lncRNA | 1 | 0 |
| TSPEAR-AS2 | lncRNA | 1 | 0 |
| DGCR12 | lncRNA | 1 | 0 |
| LINC00895 | lncRNA | 1 | 0 |
| MIAT | lncRNA | 1 | 0 |
| Z95114.1 | lncRNA | 1 | 0 |
| AL022311.1 | lncRNA | 1 | 0 |
| OGFRP1 | lncRNA | 1 | 0 |
| Z93241.1 | lncRNA | 1 | 0 |
| AL031595.3 | lncRNA | 1 | 0 |
| AL031595.2 | lncRNA | 1 | 0 |
| MIRLET7BHG | lncRNA | 1 | 0 |
| C22orf34 | lncRNA | 1 | 0 |
| FTX | lncRNA | 1 | 0 |
| GHc-362H12.3 | lncRNA | 1 | 0 |
| AL035425.3 | lncRNA | 1 | 0 |
| LINC01285 | lncRNA | 1 | 0 |
| AC245140.2 | lncRNA | 1 | 0 |
| TTTY15 | lncRNA | 1 | 0 |
| AL513497.1 | lncRNA | 1 | 0 |
| AL050341.2 | lncRNA | 1 | 0 |
| ERICH3-AS1 | lncRNA | 1 | 0 |
| AL390036.1 | lncRNA | 1 | 0 |
| RP11-640M9.1 | lncRNA | 1 | 0 |
| ASH1L-AS1 | lncRNA | 1 | 0 |
| DNM3OS | lncRNA | 1 | 0 |
| AL162431.2 | lncRNA | 1 | 0 |
| LINC01031 | lncRNA | 1 | 0 |
| MIR29B2CHG | lncRNA | 1 | 0 |
| AC132154.1 | lncRNA | 1 | 0 |
| LINC01126 | lncRNA | 1 | 0 |
| AC016747.1 | lncRNA | 1 | 0 |
| AC007878.1 | lncRNA | 1 | 0 |
| CYTOR | lncRNA | 1 | 0 |
| MIR4435-2HG | lncRNA | 1 | 0 |
| AC017002.6 | lncRNA | 1 | 0 |
| NIFK-AS1 | lncRNA | 1 | 0 |
| AC097468.3 | lncRNA | 1 | 0 |
| AC009502.1 | lncRNA | 1 | 0 |
| LIMD1-AS1 | lncRNA | 1 | 0 |
| U73169.1 | lncRNA | 1 | 0 |
| AC139887.2 | lncRNA | 1 | 0 |
| DANCR | lncRNA | 1 | 0 |
| AC010442.1 | lncRNA | 1 | 0 |
| LINC01184 | lncRNA | 2 | 0.01001 |
| AC005062.1 | lncRNA | 1 | 0 |
| AC018648.1 | lncRNA | 1 | 0 |
| SNHG15 | lncRNA | 1 | 0 |
| AC069281.2 | lncRNA | 1 | 0 |
| AC005072.1 | lncRNA | 1 | 0 |
| AC078846.1 | lncRNA | 1 | 0 |
| JHDM1D-AS1 | lncRNA | 1 | 0 |
| AL353795.3 | lncRNA | 1 | 0 |
| EBLN3P | lncRNA | 2 | 0.01001 |
| AL138756.1 | lncRNA | 1 | 0 |
| AL161908.1 | lncRNA | 1 | 0 |
| PPP1R26-AS1 | lncRNA | 1 | 0 |
| SNHG7 | lncRNA | 1 | 0 |
| H19 | lncRNA | 1 | 0 |
| SBF2-AS1 | lncRNA | 2 | 0.01001 |
| AC055860.1 | lncRNA | 1 | 0 |
| AP006333.1 | lncRNA | 1 | 0 |
| MIR194-2HG | lncRNA | 1 | 0 |
| AP000873.2 | lncRNA | 1 | 0 |
| AP001273.1 | lncRNA | 1 | 0 |
| PCBP2-OT1 | lncRNA | 1 | 0 |
| AC023509.1 | lncRNA | 1 | 0 |
| AGAP2-AS1 | lncRNA | 1 | 0 |
| LINC01619 | lncRNA | 1 | 0 |
| LINC02453 | lncRNA | 1 | 0 |
| HELLPAR | lncRNA | 1 | 0 |
| AC127164.1 | lncRNA | 1 | 0 |
| AL132780.1 | lncRNA | 1 | 0 |
| G2E3-AS1 | lncRNA | 1 | 0 |
| AL132639.2 | lncRNA | 1 | 0 |
| AC005520.2 | lncRNA | 1 | 0 |
| AL121839.2 | lncRNA | 1 | 0 |
| AC141586.5 | lncRNA | 1 | 0 |
| AC109460.3 | lncRNA | 1 | 0 |
| AC135050.6 | lncRNA | 1 | 0 |
| CRNDE | lncRNA | 1 | 0 |
| AC015883.1 | lncRNA | 1 | 0 |
| AC132872.2 | lncRNA | 1 | 0 |
| AC132938.5 | lncRNA | 1 | 0 |
| AC005391.1 | lncRNA | 1 | 0 |
| AC010503.4 | lncRNA | 1 | 0 |
| AC114271.1 | lncRNA | 1 | 0 |
| TMEM147-AS1 | lncRNA | 1 | 0 |
| AC092295.2 | lncRNA | 1 | 0 |
| AC010327.5 | lncRNA | 1 | 0 |
| AL121894.2 | lncRNA | 1 | 0 |
| AC004019.18 | lncRNA | 1 | 0 |
| AC000068.1 | lncRNA | 1 | 0 |
| AC002470.2 | lncRNA | 1 | 0 |
| AL031587.5 | lncRNA | 1 | 0 |
| Z97055.2 | lncRNA | 1 | 0 |
| AL021392.1 | lncRNA | 2 | 0.01001 |
| LINC00894 | lncRNA | 1 | 0 |
| NPPA-AS1 | lncRNA | 1 | 0 |
| AL390195.1 | lncRNA | 1 | 0 |
| MIR181A1HG | lncRNA | 1 | 0 |
| BLACAT1 | lncRNA | 1 | 0 |
| AL117350.1 | lncRNA | 1 | 0 |
| AC016907.2 | lncRNA | 1 | 0 |
| AC092155.1 | lncRNA | 1 | 0 |
| AC005538.2 | lncRNA | 1 | 0 |
| PSMD6-AS2 | lncRNA | 1 | 0 |
| LINC02035 | lncRNA | 1 | 0 |
| AC092902.2 | lncRNA | 1 | 0 |
| CCDC37-AS1 | lncRNA | 1 | 0 |
| AC083799.1 | lncRNA | 1 | 0 |
| MBNL1-AS1 | lncRNA | 1 | 0 |
| AC069224.1 | lncRNA | 1 | 0 |
| AC098864.1 | lncRNA | 1 | 0 |
| AC106744.2 | lncRNA | 1 | 0 |
| AL359715.4 | lncRNA | 1 | 0 |
| AL590617.2 | lncRNA | 1 | 0 |
| LINC00473 | lncRNA | 1 | 0 |
| AC092171.5 | lncRNA | 1 | 0 |
| AC080080.1 | lncRNA | 1 | 0 |
| AC004080.5 | lncRNA | 1 | 0 |
| AC004918.3 | lncRNA | 1 | 0 |
| AP003469.4 | lncRNA | 1 | 0 |
| AC027031.2 | lncRNA | 1 | 0 |
| CASC19 | lncRNA | 1 | 0 |
| AC083843.3 | lncRNA | 1 | 0 |
| RFX3-AS1 | lncRNA | 1 | 0 |
| AL390726.4 | lncRNA | 1 | 0 |
| AL158152.1 | lncRNA | 1 | 0 |
| AL132656.4 | lncRNA | 1 | 0 |
| CASC2 | lncRNA | 1 | 0 |
| AC129502.1 | lncRNA | 1 | 0 |
| SNHG1 | lncRNA | 1 | 0 |
| AP002336.1 | lncRNA | 1 | 0 |
| AP000560.1 | lncRNA | 1 | 0 |
| AP003392.4 | lncRNA | 1 | 0 |
| AC092828.1 | lncRNA | 1 | 0 |
| AC026124.2 | lncRNA | 1 | 0 |
| AC004812.2 | lncRNA | 1 | 0 |
| AC073857.1 | lncRNA | 1 | 0 |
| AC137767.1 | lncRNA | 1 | 0 |
| AC145423.3 | lncRNA | 1 | 0 |
| AC068790.5 | lncRNA | 1 | 0 |
| LINC00567 | lncRNA | 1 | 0 |
| LINC00641 | lncRNA | 1 | 0 |
| AL079343.1 | lncRNA | 1 | 0 |
| AL162171.3 | lncRNA | 1 | 0 |
| AC124312.3 | lncRNA | 1 | 0 |
| AC013652.1 | lncRNA | 1 | 0 |
| AC016134.1 | lncRNA | 1 | 0 |
| AC009269.5 | lncRNA | 1 | 0 |
| AC013489.1 | lncRNA | 1 | 0 |
| AC087284.1 | lncRNA | 1 | 0 |
| AC109597.2 | lncRNA | 1 | 0 |
| AC106739.1 | lncRNA | 1 | 0 |
| AC135048.1 | lncRNA | 1 | 0 |
| AC007906.1 | lncRNA | 1 | 0 |
| AC136621.1 | lncRNA | 1 | 0 |
| AC020978.4 | lncRNA | 1 | 0 |
| LINC02001 | lncRNA | 1 | 0 |
| AC125257.1 | lncRNA | 1 | 0 |
| AC103702.1 | lncRNA | 1 | 0 |
| AC015909.3 | lncRNA | 1 | 0 |
| AC004687.1 | lncRNA | 1 | 0 |
| AC005746.1 | lncRNA | 1 | 0 |
| SNHG20 | lncRNA | 1 | 0 |
| BAIAP2-AS1 | lncRNA | 1 | 0 |
| AC145207.5 | lncRNA | 1 | 0 |
| DLGAP1-AS1 | lncRNA | 1 | 0 |
| AC091060.1 | lncRNA | 1 | 0 |
| AC011444.1 | lncRNA | 1 | 0 |
| AC020928.1 | lncRNA | 1 | 0 |
| AC011468.1 | lncRNA | 1 | 0 |
| AL049712.1 | lncRNA | 1 | 0 |
| AL035661.1 | lncRNA | 1 | 0 |
| AL121832.3 | lncRNA | 1 | 0 |
| BTG3-AS1 | lncRNA | 1 | 0 |
| TUG1 | lncRNA | 1 | 0 |
| AC004656.1 | lncRNA | 1 | 0 |
| AC244197.2 | lncRNA | 1 | 0 |
